# An All-Fiber Optical Sensor Combined with FBG and CFBG-FP for Attitude Estimation

**DOI:** 10.3390/s22155562

**Published:** 2022-07-26

**Authors:** Yifan Li, Shuidong Xiong, Ji Xia

**Affiliations:** 1 College of Meteorology and Oceanography, National University of Defense Technology, Changsha 410073, China; liyifan2512@nudt.edu.cn (Y.L.); xiong_shuidong@nudt.edu.cn (S.X.); 2 Hunan Key Laboratory for Marine Detection Technology, Changsha 410073, China

**Keywords:** sensor, fiber Bragg grating, chirped grating, Fabry-pérot cavity, magnetostriction, attitude angle

## Abstract

In order to meet the needs of attitude correction of the optical fiber sensor array, an all-fiber optical sensor combined with FBG and CFBG-FP is proposed to monitor its attitude angle. The FBG sensor for pitch and roll angles detection employs a geometric model solution to shift the center wavelength of the three fiber Bragg gratings connected to it through the mass sphere, allowing for pitch and roll angle detection. Based on the magnetostrictive effect, the CFBG-FP sensor for heading angle detection employs changes of the cavity length of the chirped grating Fabry-Pérot cavity connected to its surface. The method of interpolation data processing and derivation of the attitude angle matrix is used to realize heading angle detection. The experimental results indicate that the FBG sensor for the pitch and roll angles detection has an effective detection range of 180 degrees and an angle measurement error of less than 1.5 degrees. Meanwhile, in the region beyond 10 degrees in the geomagnetic north-south direction, the angle measurement error of the CFBG-FP sensor for the heading angle detection is less than 1.3 degrees. Moreover, the impact of temperature in the system is minimized, demonstrating the validity of this combined fiber optical approach in the attitude angle measurements.

## 1. Introduction

The maritime environment is complicated and evolving, and while the underwater sensing system is operating, attitude changes of the measurement system such as pitching and overturning will occur. In order to determine the orientation of the underwater detection system in the geographic coordinate system, the attitude sensor is usually installed on the underwater detection system. The existing attitude sensors mostly adopt satellite navigation systems, inertial sensors and magnetic heading sensors [1,2,3]. However, the marine environment has an obvious inhibitory effect on the transmission of electromagnetic waves [4]. At present, the attitude acquisition devices used in astronomical navigation systems and satellite navigation systems [5,6,7], which are commonly utilized in ground and aviation systems, are challenging to employ in underwater systems. It should be noted that inertial sensors are able to carry out attitude estimation. As a high-performance inertial sensor, the gyroscope (for example, laser gyroscope and fiber optic gyroscope) is used to measure the angular velocity of the carrier, but it has some technical limits, such as high cost and large volume, which impedes its large-scale engineering application. It has benefited from the introduction of micro-electro-mechanical system (MEMS) technology and it is widely promoted for the implementation and development in miniature navigation systems [8,9,10]; the volume of the MEMS-based gyroscope is smaller than that of the laser gyroscope or fiber optic gyroscope. The MEMS-based inertial sensor is vulnerable to the influence of temperature and unable to be directly used in all cases. In addition, there are also large errors that accumulate over time, i.e., the outputs by MEMS-based inertial sensor, and hence, it must be calibrated and compensated. Finally, as a common magnetic heading sensor, the electronic compass [11,12,13] realizes the heading angle measurement of the underwater system, but it is easily affected by environmental factors and has a slow response. For these reasons, these sensors cannot always exhibit stable performance with regard to attitude estimation.

Compared with the methods that only use one type of sensor, the combined detection method can overcome various measurement errors and improve measurement accuracy. The MARG (magnetic, angular rate, and gravity) sensing system [14,15,16] is one of the various combined detection sensors for acquiring the attitude angle of the underwater detection system. It comprises magnetic sensors, a gyroscope, and an accelerometer, and it can also be seen as a combination of a magnetic sensor and inertial measurement unit (IMU). Markus et al. [17] proposed a low-cost nine degree of freedom MARG sensor system to measure the attitude angle with an average root mean square error of fewer than 3.89 degrees. Jurman et al. [18] used low-cost MEMS and Anisotropic Magneto-Resistive (AMR) sensors to measure the attitude angle with an average root mean square error of less than 1.2 degrees through the adaptive Kalman filter data fusion technology. Since the output of the MARG sensor system is a digital signal, it is easily disrupted during long-distance transmission. Meanwhile, the loss of the signal should be addressed for the reason that it is not conducive to the long-distance detection of the underwater detection system.

In order to realize the all-fiber structure of the signal detection and transmission, it is urgent to develop a low-cost, small-size, low-power, and high-precision attitude measurement combined sensor. It can withstand an underwater system with the capacity to operate safely and accurately measure and correct attitude. Compared with the electrical attitude sensor, the attitude sensor based on optical fiber for sensing and signal transmission is characterized by a wide frequency band, high sensitivity, compact size, strong anti-interference ability, suitability for underwater applications, easily formed large-scale arrays, etc. [19].

To achieve high precision and reliability in the optical fiber attitude sensor, it is critical to design and manufacture the transducer in a reasonable manner. Chen et al. [20] designed an inclination sensor based on fiber Bragg gratings (FBG), which is insensitive to temperature. It is composed of an FBG and a weight block. Within the measurement range of ±15 degrees, the measurement accuracy can reach ±0.167 degrees; however, only one angle can be recorded and the dynamic range is limited. Au et al. [21] proposed a system with four FBGs linked by one weight block. Within the measurement range of ±35 degrees, the measurement accuracy can reach ±0.051 degrees, realizing the measurement of pitch angle and roll angle. The dynamic range is further improved, but the stability of the system is reduced by using the cycloid suspension structure. Zhang et al. [22] developed a full-polarization maintaining optical fiber weak magnetic field sensor based on rare earth giant magnetostrictive materials. The angle measurement accuracy with the Michelson interferometer is greater than 1.3 degrees beyond the geomagnetic north-south direction of 10 degrees. However, only the heading angle can be measured and the local geomagnetic field intensity must be estimated beforehand.

In order to address the aforementioned issues, this paper proposes a combined sensor that achieves all-fiber detection of the full attitude estimation. The combined sensor is composed of an optical fiber inclination sensor and optical fiber magnetic sensor based on magnetostrictive material. The impact of temperature on measurement is eliminated thanks to the construction of the combined sensor, and the attitude angle measurement in a wide dynamic range is realized. Furthermore, the measurement accuracy and stability of the system are improved with a minimal reduced volume. The combined sensor is easy to be integrated with the existing optical fiber sensing system, which is of great significance for the attitude monitoring of remote detection systems.

## 2. Structural Design and Method for Attitude Estimation

### 2.1. Design and Fabrication of an Attitude Measurement System

**Figure 1:**
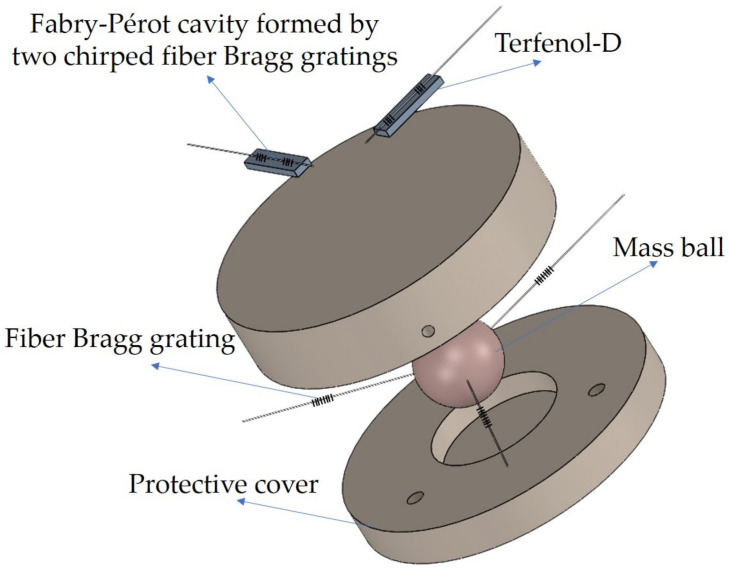
Overall schematic diagram of the all-fiber optical sensor combined with FBG and CFBG-FP.

As shown in Figure 1, the combined sensor is composed of two parts. One part includes a mass ball and three FBGs for the pitch and roll angle detection. The other part includes two magnetostrictive material sheets placed orthogonally and an optical fiber with a Fabry-Pérot cavity consisting of two chirped fiber Bragg gratings (CFBG-FP) positioned along its length direction for the heading angle detection. The function of the polymer polyethylene pipe is to keep the mass ball’s movement range under control. As a result, the pulling force of the mass ball on the FBG is constantly under axial extension to avoid excessive pulling power, which prevents the FBG from being fatigued. Terfenol-D [23] is the magnetostrictive material, which possesses superior magnetic characteristics and energy conversion efficiency than pure nickel (Ni) and piezoelectric ceramics (PZT).

In the manufacturing of the FBG sensor for the pitch and roll angles detection, one end of the three FBGs is attached to the mass ball, and the other end is bonded to the polymer polyethylene pipe with a certain axial tension. As a result, the FBG can work under the tension load to reduce the measurement error. To avoid the grating from directly bearing the bulk of the sphere and considerable displacement, the FBG is encased in a capillary steel tube. The measuring range and stability of pitch and roll angles are substantially enhanced with this design, making it superior to other similar sensors. The CFBG-FP sensor for heading angle detection is subjected to adequate stress throughout the manufacturing process, in order to create a fiber with a tensional condition. Subsequently, epoxy glue is used to adhere the fiber with the CFBG-FP region to the magnetostrictive sheet. As a result, the deformation coupling efficiency between the magnetostrictive sheet and the Fabry-Pérot cavity is improved. In the demodulation of the heading angle, the two magnetostrictive materials are placed orthogonally, which overcomes the error caused by the need to introduce the local magnetic field strength for the same type of sensor.

**Figure 2:**
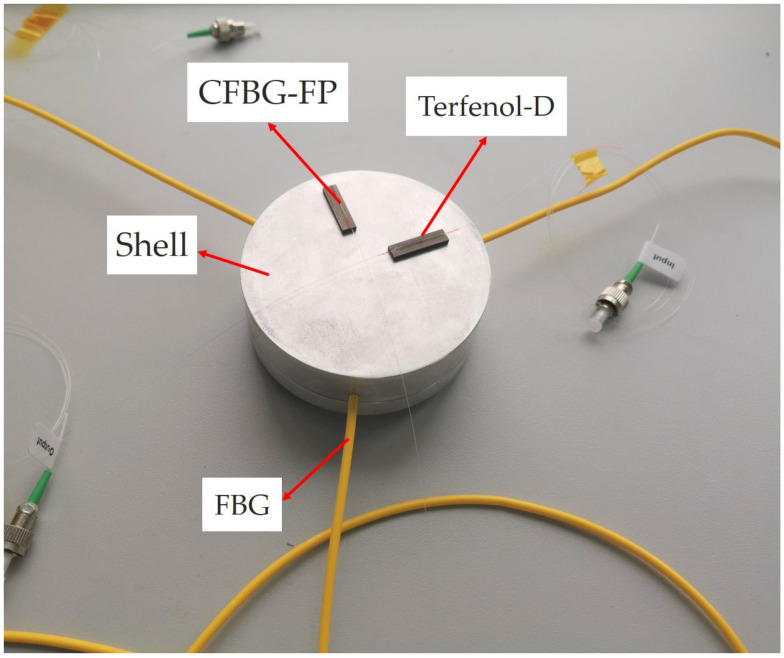
Experimental sensing unit of all-fiber optical sensor combined with FBG and CFBG-FP.

The protective cover of the combined sensor is a polymer polyethylene pipe with an outer diameter of 82 mm, an inner diameter of 34 mm, and a height of 26 mm. There are polymer polyethylene sealing bases matching the polymer polyethylene pipe at the top and bottom. Three holes with a diameter of 2 mm and a depth of 24 mm are drilled at 120 degrees off the side of the polymer polyethylene pipe. Meanwhile, three holes with a diameter of 2 mm and a depth of 5 mm are drilled of the mass ball, and each is separated 120 from the others. The characteristic parameters of the FBG sensor for pitch and roll angles detection are shown in Table 1. The Terfenol-D sheet used in the CFBG-FP sensor for heading angle detection is 20 mm long. In order to exhibit the better interference effect with regard to the accuracy that can be achieved, the CFBG-FP is designed for the heading angle detection. The CFBG-FP consists of two chirped gratings with a center wavelength of 1550 nm, a reflectivity of 10%, a grating length of 8 mm, a chirped coefficient of 6.5 nm/cm, and a grating interval of 5 mm. The physical drawing of the combined sensor is shown in Figure 2.

### 2.2. Sensing Principles of the FBG Sensor for Pitch and Roll Angles Detection

**Figure 3:**
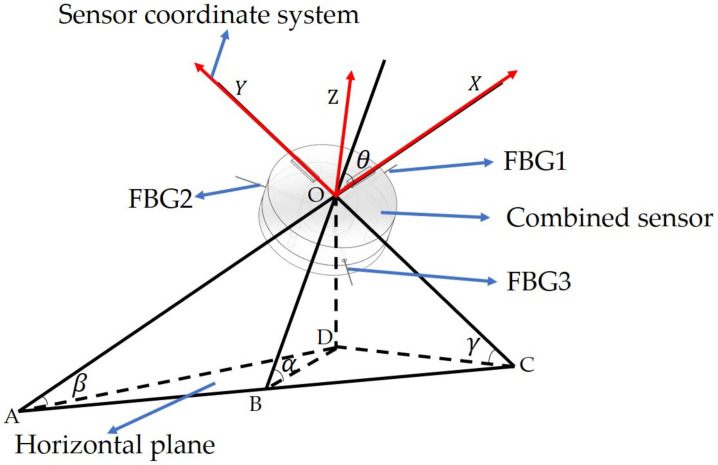
Schematic diagram of pitch and roll angle of the sensor coordinate system with the horizontal plane. The three FBGs are marked as FBG1, FBG2, and FBG3, respectively. The X-axis is the mounting direction of FBG1. In the platform plane, the Y-axis represents the direction of FBG1 rotated 90 degrees counterclockwise. The Z-axis is perpendicular to the plane of the combined sensor. *α* is the angle between the plane of the combined sensor and the horizontal plane. *θ* is the angle between the X-axis and line OB. *β* (pitch angle) is the angle between the X-axis and the horizontal plane. *γ* (roll angle) is the angle between the Y-axis and the horizontal plane.

The FBG is a structure inscribed into the core of an optical fiber, where the core refractive index is cyclical variability which works as a grating. Once a broad band spectrum light transmitted into FBG, only a small amount of light is back-reflected. The reflected light is centered at the Bragg wavelength, which can be used for sensing (such as strain, temperature, and acceleration). The variations of the pitch angle and roll angle can be resolved through processing outputs of FBGs (see Figure 3).

In fact, the FBG sensor for pitch and roll angles detection is an inertial sensor (accelerometer). According to the force balance, the radial force received by the three FBGs can be obtained as:

(1) $$\left[\begin{array}{c} \mathrm{mg}\sin\alpha \\ 0\\ 0 \end{array}\right]=\left[\begin{array}{ccc} \cos\theta & \cos\left(\frac{2}{3}\pi -\theta \right) & -\cos\left(\frac{\pi }{3}-\theta \right)\\ \sin\theta & -\sin\left(\frac{2}{3}\pi -\theta \right) & \sin\left(\frac{\pi }{3}-\theta \right)\\ 0 & 0 & 0 \end{array}\right]\left[\begin{array}{c} F1\\ F2\\ F3 \end{array}\right],$$

where $F_{i}$ is the radial force received by the FBG and *i* is the FBG serial number.

According to the material mechanics analysis, the strain ($\varepsilon_{i}$) at the cross-section area of the fiber (*S*) on the FBG [24] can be obtained as $\varepsilon_{i}=F_{i}/ES$. Three FBGs are operated in the same temperature field, and the Bragg wavelength ($\lambda_{B}$) is shifted due to changes in strain ($\varepsilon_{i}$) and temperature (ΔT). The relationship between the FBG wavelength shift with strain and temperature can be expressed as [25]:

(2) $$\Delta \lambda_{i}=\lambda_{B}(1-P_{e})\varepsilon_{i}+\lambda_{B}(\vartheta +\xi )\Delta T,$$

where $\Delta \lambda_{i}$ is the variation of the central wavelength of FBG, $P_{e}$ is effective photo-elastic coefficient, ϑ is the thermal expansion coefficient of the fiber, and ξ is the hermo optical coefficient of the fiber.

Focusing on applications of pitch and roll angles detection, when each FBG is attached to the mass ball, any deformation occurring on the fiber is directly transmitted to the FBG. As a consequence, the displacements of mass ball produce variations in the out-put of FBG, resulting in $\Delta \lambda_{i}$. Thus, the relationship between the center wavelength shift of the three FBGs and β (Pitch angle) can be obtained as:

(3) $$2\Delta \lambda_{1}-\Delta \lambda_{2}-\Delta \lambda_{3}=\frac{8mg\lambda_{B}(1-P_{e})}{E\pi d^{2}}\sin\beta =K_{pitch}\sin\beta ,$$

Similarly, the relationship between the center wavelength shift of the three FBGs and γ (roll angle) can be obtained as:

(4) $$\Delta \lambda_{2}-\Delta \lambda_{3}=\frac{8mg\lambda_{B}(1-P_{e})}{\sqrt{3}E\pi d^{2}}\sin\gamma =K_{roll}\sin\gamma ,$$

where E is the Young’s modulus of optical fiber, d is the diameter of bare fiber, $K_{pitch}$ is the pitch angle sensing coefficient, and $K_{roll}$ is the roll angle sensing coefficient.

Due to the differential compensation method [26] is adopted, it can be seen from Equations (3) and (4) that this method eliminates the influence of ambient temperature on attitude angle measurement. By adjusting the weight of the mass ball, the angle sensing coefficient can be adjusted. There is a linear relationship between the difference of the center wave-length shifts and the sine of the angle. According to the parameters shown in Table 1, the theoretical sensing coefficients of pitch and roll angles are 2.3445 nm ($K_{pitch}$) and 1.3536 nm ($K_{roll}$), respectively.

### 2.3. Sensing Principles of the Fiber CFBG-FP Sensor for Heading Angle Detection

**Figure 4:**
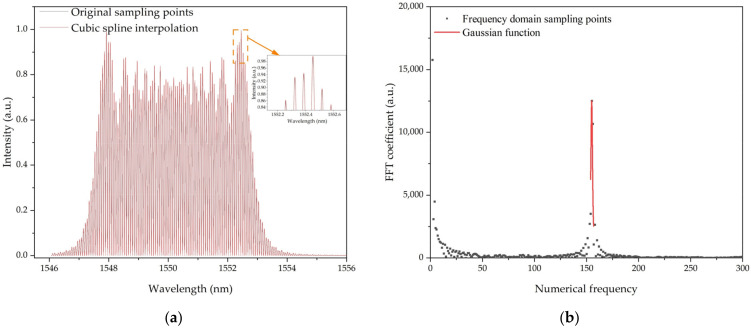
Simulated results of CFBG-FP. (**a**) Cubic spline interpolation results and original sampling points of the interference light intensity. (**b**) Amplitude frequency response characteristic of the interference light intensity.

The CFBG-FP is an optical fiber with a Fabry-Pérot cavity consisting of two chirped fiber Bragg gratings. Once a broad band spectrum light transmitted into CFBG-FP, only a small amount of light is back-reflected. The light waves are in interference in this CFBG-FP cavity with the CFBG. By measuring interference light intensity, the corresponding length of cavity can be calculated. Compared with the other FP cavity, CFBG-FP has wider spectral range, richer spectral information, and more resonant peaks, so the phase sensitivity of signal processing is very high, as shown Figure 4.

**Figure 5:**
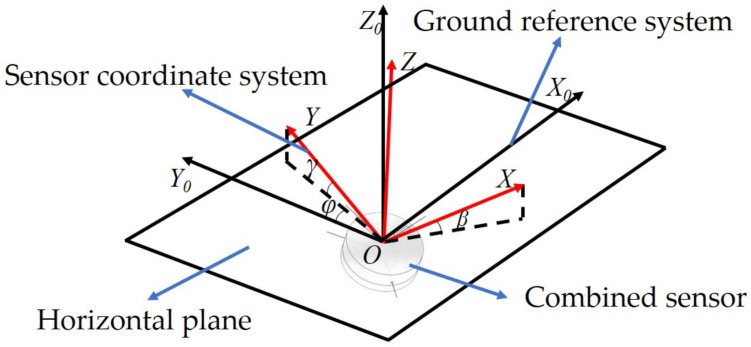
Diagram of the relationship between the sensor coordinate system and the ground reference system. The *XYZ* coordinate axis is the coordinate system of the combined sensor, and *X*_0_*Y*_0_*Z*_0_ coordinate axis is the ground reference system. *β*, *γ*, *φ* are the pitch angle, roll angle, and heading angle, respectively.

In the CFBG-FP sensor for heading angle detection, two optical fibers with CFBG-FPs are orthogonally mounted on the polymer polyethylene pipe of mass ball, and the plane of the two CFBG-FPs is parallel to the platform plane. One of the optical fibers with CFBG-FP is positioned in the same direction as FBG1. In the initial state, the value of heading angle is 0 degrees. When the attitude of the combined sensor changes, the magnetostrictive material deforms under the effect of the geomagnetic field, causing the cavity length of the CFBG-FP to vary. The change of attitude is shown in Figure 5.

The magnetostrictive strain is caused by the magnetostrictive sheet (Terfenol-D) under the action of the geomagnetic field. The relationship between the strain and the magnetic field strength (*H*) [27] can be expressed as $\varepsilon =CH^{2}$. When Terfenol-D sheet is operated in the linear magnetic strain region, the relationship between the change of cavity length and the magnetic field can be expressed as:

(5) $$\varepsilon =\frac{\Delta l}{l_{0}}=C_{\mathrm{eff}}H,$$

where Δl is the variation of cavity length, $l_{0}$ is the initial cavity length, and $C_{eff}$ is the equivalent magnetostrictive coefficient.

In the demodulation of cavity length, the main steps include interference spectrum acquisition, frequency-domain signal interpolation processing, discrete Fourier transform, and frequency domain interpolation correction.

Considering the optical spectrum analyzer samples the wavelength at equal intervals, the received light intensity should be interpolated before the discrete Fourier transform [28] in the frequency domain [29]. Under the influence of the fence effect, the fundamental frequency of the spectrum distribution after the discrete Fourier transform is an integer. It is necessary to interpolate and correct the fundamental frequency [30] to obtain a high-precision fundamental frequency.

The relationship between the matrix [*H*_0_ 0 0]*^T^* of the magnetic field in the geographic coordinate system and the matrix [*H*_1_ *H*_2_ *H*_3_]*^T^* of the geomagnetic field in the coordinate system of the combined sensor can be expressed as:

(6) $$\left[\begin{array}{c} H_{1}\\ H_{2}\\ H_{3} \end{array}\right]=\left[\begin{array}{ccc} \cos(\gamma )\cos(\varphi )+\sin(\gamma )\sin(\beta )\sin(\varphi ) & \sin(\gamma )\sin(\beta )\cos(\varphi )-\cos(\gamma )\sin(\varphi ) & \sin(\gamma )\cos(\beta )\\ \cos(\beta )\sin(\varphi ) & \cos(\beta )\cos(\varphi ) & -\sin(\beta )\\ \cos(\gamma )\sin(\beta )\sin(\varphi )-\sin(\gamma )\cos(\varphi ) & \sin(\gamma )\sin(\varphi )+\cos(\gamma )\sin(\beta )\cos(\varphi ) & \cos(\gamma )\cos(\beta ) \end{array}\right]\left[\begin{array}{c} H_{0}\\ 0\\ 0 \end{array}\right]$$

Focusing on applications of heading angle detection, when each CFBG-FP is attached to the magnetostrictive sheet, any variation occurring on the magnetic field strength (H) is directly transmitted to the magnetostrictive sheet, and in turn, to the CFBG-FP. As a consequence, the cavity length produces variations in the output of CFBG-FP, resulting in Δl. The relationship between φ (heading angle) and demodulated cavity length can be obtained as:

(7) $$\tan\varphi =\frac{\left(l_{2}-l_{0}\right)\cos(\gamma )}{\left(l_{1}-l_{0}\right)\cos(\beta )-\left(l_{2}-l_{0}\right)\sin(\gamma )\sin(\beta )},$$

where $l_{1}$ and $l_{2}$ are the cavity lengths of the two CFBG-FPs, respectively. It can be seen that the relative variation of the cavity length is used while solving the heading angle, which eliminates the influence of temperature on the angle measurement, exactly as what has been done when measuring pitch and roll angle.

## 3. Experimental Results

### 3.1. Experimental System

**Figure 6:**
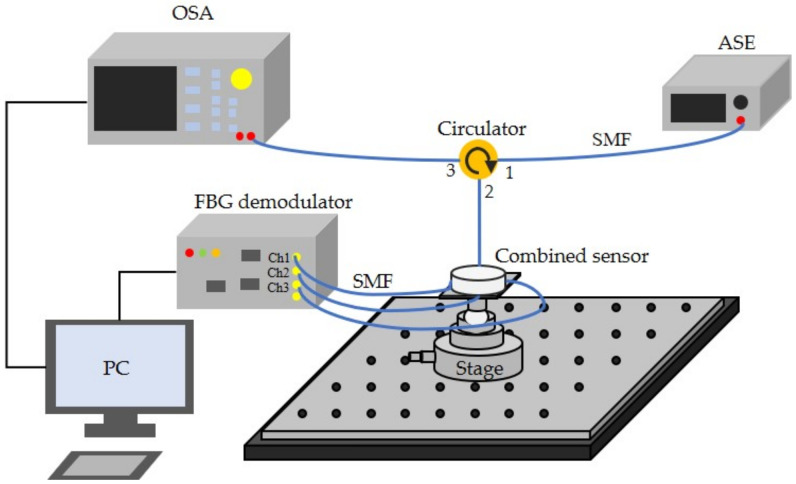
Experimental setup for the attitude measurement system.

The attitude angle measurement system is shown in Figure 6. The beams output from the three channels of the fiber Bragg grating demodulator (SM130, Micron Optics Inc., Atlanta, GA, USA) are transmitted to FBG1, FBG2, and FBG3 through single-mode fiber (SMF) for strain detection. When the attitude of the combined sensor changes, the length of the FBG changes due to the micro movement of the mass ball, and the optical signal in the fiber is modulated. The reflected light returns to the original path and is detected by the FBG demodulator. The change of the central wavelength of the three FBGs can be obtained, and the variations of the pitch angle and roll angle can be resolved through computer processing. At the same time, the light beam from the amplified spontaneous emission (ASE-C-11-G, Hoyatek Co., Ltd., Shenzhen, China) is injected into the optical circulator through port1 and transmitted to the CFBG-FP for magnetic field detection. The geomagnetic field changes the length of the magnetostrictive sheet and then changes the length of the FP cavity fixed on its surface. The optical signal in the optical fiber is modulated. by the variation of the FP cavity length, the reflected light passes through port 2 to port 3 and is finally detected by the optical spectrum analyzer (AQ6370 OSA, YOKOGAWA, Tokyo, Japan, with a wavelength resolution of 0.02 nm). By combining the data acquired by OSA with the known pitch and roll angle readings, the heading angle can be calculated by computer processing.

The FBG sensor for pitch and roll angles detection and the CFBG-FP sensor for heading angle detection are fixed on an adjustable stage. The angle of the adjustable stage is changed from −90 degrees to 90 degrees to cover the *β* range that could be applied to the sensing system to determine the pitch angle. Values of *β* are collected at a step size of 5 degrees, while the outputs of FBGs (i.e., $\Delta \lambda_{i}$) are recorded by a FBG demodulator. In addition, other angles are unchanged. As references of an adequate adjustment, an inclinometer (LM320B, UNI-T Inc., Shenzhen, China), is mounted on the stage, at the same time. The whole test sustains about 30 s for each data set, so the stability of the measured angle detecting has a negligible influence on the output of the FBG sensor for pitch and roll angles detection.

**Figure 7:**
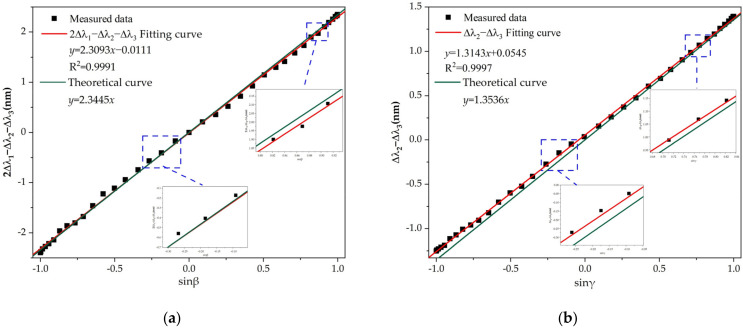
The measured relationship of the central wavelength shift vs. the sine of the attitude angle. (**a**) Central wavelength shift vs. the sine of the pitch angle. (**b**) Central wavelength shift vs. the sine of the roll angle.

Figure 7a indicates the linear relationship between the shift of center wavelength shifts vs. the sine of the pitch angle. The high correlation coefficients R^2^ > 0.99 demonstrates a good agreement between the experimental data and the linear fitting. The experimental method for roll angle is the same as that of detection for pitch angle. In Figure 7b, the linear relationship between the difference of the center wavelength shifts vs. the sine of the roll angle is obtained with a linearity coefficient greater than 0.999. The different sensing coefficient values with theoretical coefficient values are attributed to the imperfect fabrication process, which is manually executed. Certainly, despite the fact that manufacturing defects may be reduced or even eliminated, it is inevitable that the capillary steel tube used to encapsulate the fiber Bragg grating carries a portion of strain of the mass ball on the FBG. In fact, the contribution of strain carried by the capillary steel tube on estimation of the pitch and roll angles may be deemed negligible. At the same time, it can also be seen that some measured data points deviate from the fitting curve, which is caused by ambient noise. As shown in Figure 7, when the angle changes within ±50 degrees, the variation of the central wavelength is small with an angle measurement error of greater than 1 degree. Beyond this angle range, the variation of the central wavelength is large with an angle measurement error of less than 1 degree. The small angle measurement error is due to the fact that the tension change of each FBG at a small angle is so subtle, resulting in the shift of the center wavelength induced by external disturbances (vibration, noise, etc.) more prominent. Although external disturbances are inevitable, the high values of correlation coefficients (R^2^ > 0.99 for pitch and roll angles detection) ensured agreement between the experimental data and the linear model.

**Figure 8:**
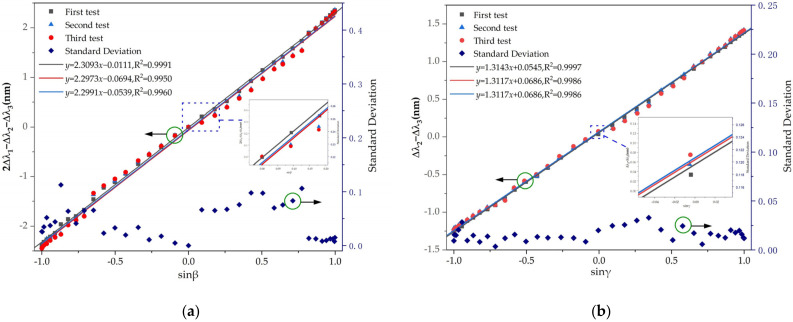
Multiple measured relationship of the central wavelength shift and standard deviation vs. the sine of the attitude angle. (**a**) Central wavelength shift and standard deviation vs. the sine of the pitch angle. (**b**) Central wavelength shift and standard deviation vs. the sine of the roll angle.

The measurement of pitch angle is repeated three times with the test angle varying from −90 degrees to 90 degrees. The relationship between the value of 2∆λ_1_−∆λ_2_−∆λ_3_ vs. the sine of the pitch angle for the sensor throughout the three experiments is shown in Figure 7a. Under the same conditions, repeatability indicates the degree of inconsistency of fitting curves of multiple measurements, which can be obtained as $e_{z}=c_{k}\sigma_{\max}/y_{FS}$. The full scale of the central wavelength shift ($y_{FS}$) is 4.7682 nm (see Figure 8a). Here, σ is the standard deviation obtained under Bessel conditions. It can be obtained from Figure 8a that the maximum value of standard deviation ($\sigma_{\max}$) is 0.11244 nm. When the confidence probability is 99.7% ($c_{k}=3$), the repeatability error ($e_{z}$) of the three tests is 7.07%, indicating that the measurement of the pitch angle has good repeatability. Similarly, the test for the roll angle is also conducted three times from −90 degrees to 90 degrees. The relationship between the value of ∆λ_2_−∆λ_3_ vs. the sine of the roll angle for the sensor throughout the three tests is shown in Figure 8b. When the confidence probability is 99.7%, the repeatability error of three tests is 3.72%, indicating that the measurement of the roll angle has good repeatability. As shown in Figure 8a, because of the shift of the center wavelength induced by external disturbances (vibration, noise, etc.), some values of standard deviation are greater than 0.025 nm. The high correlation coefficients R^2^ > 0.99 demonstrates a good agreement between the experimental data and the linear fitting in second and third tests. The acquired results are consistent across multiple tests using this sensor, demonstrating the reliability of this attitude angle measurement method.

Moreover, an experimental test is performed to confirm the viability of heading angle detection. To cover the *φ* range that the sensor may experience in reaction to the heading angle, the angle of the adjustable stage is increased from 0 degrees to 360 degrees. Values of angle *φ* are recorded at a step angle of 10 degrees, while the outputs of CFBG-FP sensors are monitored by the OSA. In the meanwhile, the experiments are conducted at a fixed horizontal angle. The cavity length of CFBG-FP sensor can be calculated through Equation (5) at a certain angle. One of CFBG-FP in the CFBG-FP sensor for heading angle detection that is placed in the same direction as FBG1 is marked as CFBG-FP1, and the other is marked as CFBG-FP2. Referring to Equation (7), the measured heading angle can be solved.

**Figure 9:**
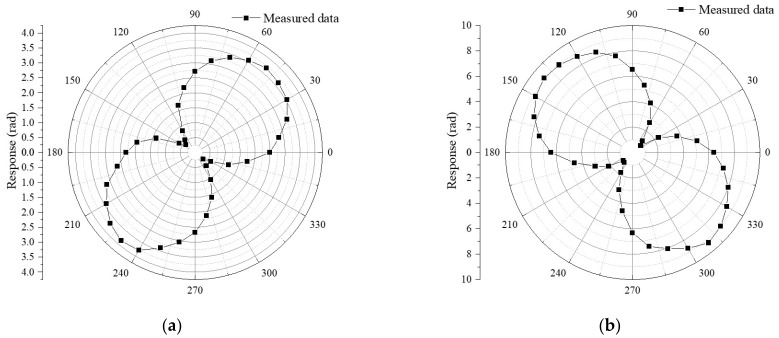
Directivity pattern of the phase response vs. the angle of the adjustable stage. (**a**) The value of the phase response of CFBG-FP1 vs. the angle of the adjustable stage. (**b**) The value of the phase response of CFBG-FP2 vs. the angle of the adjustable stage.

**Figure 10:**
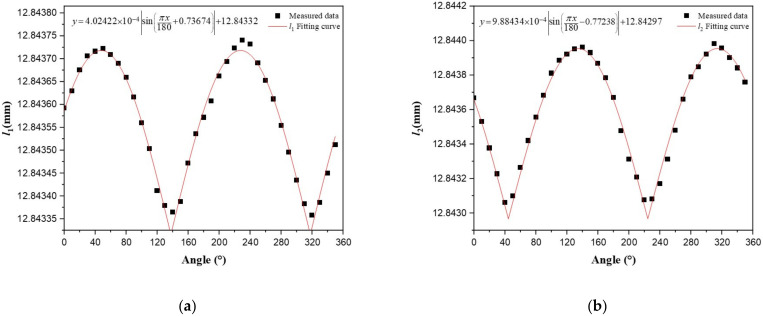
The measured relationship of cavity length of CFBG-FP vs. the angle of the adjustable stage. (**a**) Cavity length of CFBG-FP1 vs. the angle of the adjustable stage. (**b**) Cavity length of CFBG-FP2 vs. the angle of the adjustable stage.

As can be seen from Figure 9, two circles in the sketched directivity pattern have almost the same diameters. Certainly, in addition to the geomagnetic field component, other disruptive magnetic field components in the environment also have an impact on the measured phase response to magnetic field component. These unpreferable magnetic field components are mostly raised from numerous experimental devices. The geomagnetic environment around the CFBG-FP sensor could be also be disturbed by the magnetic metals, which will eventually affect how the combined sensor measures its orientation. The resolved relationship between the calculated cavity length and the angle is shown in Figure 10. According to Figure 10a, the initial phase of CFBG-FP1 is 0.73674 rad, which indicated that the included angle between the initial angle and geomagnetic north is 42.2 degrees. In addition, the initial phase of CFBG-FP2 is −0.77238 rad, which indicated that the included angle between the initial angle and geomagnetic north is −44.3 degrees. Comparing these two initial phases, it can be concluded that two orthogonal CFBG-FPs have a deviation of 3.5 degrees. The deviation of 3.5 degrees is attributable to the fabrication process, which is manually executed. Despite these errors, the results of experiments substantiate the reliability of this attitude angle measurement method. Comparing Figure 10a,b, the sensing coefficient of CFBG-FP2 is higher than that of CFBG-FP1. The strain coupling efficiency of the CFBG-FP and the magnetostrictive sheet are inconsistent, because the optical fiber is not stretched when it is attached to the magnetostrictive sheet. Fortunately, this can be overcome because all experimental data should be normalized here.

The attitude angle values measured by the CFBG-FP sensor for heading angle detection is calculated, and the comparison with the reference values is shown in Table 2. When the combined sensor rotates from north to west, the response of CFBG-FP1 decreases, and the response of CFBG-FP2 decreases first and then increases. On the contrary, when the combined sensor rotates from north to east, the response of CFBG-FP1 also decreases, and the response of CFBG-FP2 first increases and then decreases. Thus, the impact of problem with directionality on measurement (see Figure 10) is eliminated thanks to the deviation of 3.5 degrees of orthogonal CFBG-FP, and the attitude angle measurement in a wide dynamic range from east to west is realized. It can be seen that when the actual angle is more than 10 degrees to the left and right in the geomagnetic north direction, the heading angle error measured by the system is basically kept at about 1.3 degree. It can be obtained that the pitch angle and roll angle are not 0 degrees, which also explains the slight difference in the diameters of the two circles in the sketched directivity pattern (see Figure 9).

### 3.2. Potential Applications of All-Fiber Optical Sensor Combined with FBG and CFBG-FP for Attitude Estimation of Underwater Detection System

Large constructions (off-shore platforms, underwater acoustic arrays, etc.) must have sensors installed in strategic locations to establish a detection network for nondestructive testing and attitude determination. Thus, there are typically dozens or even hundreds of sensors involved. Multiplexing technology [31] is used to simplify the complexity of the system and utilize the same system to demodulate the measurement data from many sensors. The optical fiber sensor has greater benefits than the traditional sensor because multiplexing technology ensures favorable measurement accuracy and reliability.

**Figure 11:**
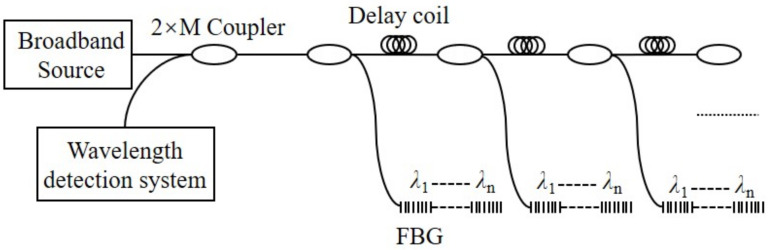
FBG sensor multiplexing technology: WDM/TDM.

**Figure 12:**
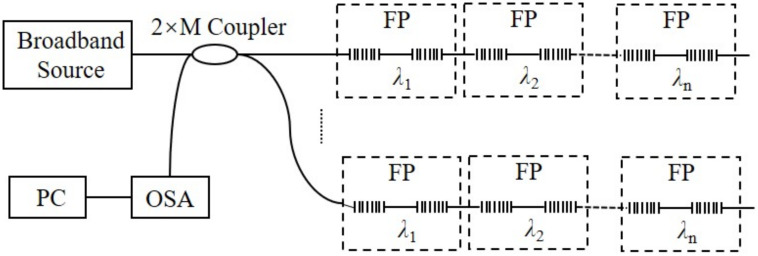
CFBG-FP sensor multiplexing technology: WDM/FDM.

Multiplexing is a way to transmit multiple sensing signals through a single optical fiber or a pair of optical fibers, which can improve the channel capacity and reduce the transmission cost of the system. The FBG sensor for pitch and roll angles detection is composed of FBG. When wavelength division multiplexing (WDM) is adopted, the measurement range of a single FBG and the bandwidth of the light source determine the number of multiplexes. However, by using wavelength division/time division multiplexing (TDM), a large number of FBG arrays can be realized, as shown in Figure 11. The CFBG-FP sensor for heading angle detection is composed of CFBG-FPs. When frequency division multiplexing (FDM) is used, the number of fibers FP array multiplexing is limited by the sampling theorem and spectral resolution. However, when wavelength division/frequency division multiplexing is adopted, the multiplexing number of sensors can be greatly increased, as shown in Figure 12.

The fiber optical underwater acoustic detection array also adopts multiple multiplexing methods to form an array [32]. By reasonably designing the cavity length of CFBG-FP and the length of FBG, the number of sensors can be increased, which makes it easier to integrate into the existing detection system, and realizes the three-dimensional attitude angle measurement of large-scale underwater acoustic detection array.

## 4. Conclusions

In this paper, an all-fiber optical sensor combined with FBG and CFBG-FP is proposed for attitude estimation which can be potentially integrated into the underwater optical fiber sensing system. In the attitude estimation method, the FBG sensor is effective for detecting the pitch and roll angles, while the CFBG-FP sensor is functional for heading angle measurement. Compared with the ordinary fiber Bragg grating sensor, the combined sensor increases the angle measurement range [20,21]. The range of pitch angle and roll angle is −90 degrees to 90 degrees, and the measurement error is within 1.5 degrees. When the measurement angle of the heading angle is beyond 10 degrees in the geomagnetic north-south direction, the accuracy error is within 1.3 degrees. It should be noted that the theoretical analysis indicates that optimization of optical fiber sensor features, such as weight of mass ball and magnetostrictive material type, is beneficial to improving the sensitivity of the combined sensor. Besides, the proposed attitude estimation method adopts the self-compensation method to eliminate the influence of temperature on the measurement of the pitch angle, roll angle, and heading angle. As a result, preliminary experiment results show that the proposed combined sensor has good stability and repeatability. Moreover, the combined sensor adopts the all-fiber optical sensing configuration for the measurement of the pitch angle, roll angle, and heading angle, which can be potentially integrated into the optical fiber sensing system based on the multiplexing technology. The combined sensor could also be used to track the attitude of long-distance detection systems and can potentially satisfy the self-attitude measurement requirements of marine geological survey and underwater acoustic detection systems.

## Figures and Tables

**Table 1:** Characteristic parameters of the FBG sensor for the pitch and roll angles detection.

The Size Specifications and Symbol	Quantity	Value
*λ*_1_	Center wavelength of the FBG1	1549.9987 nm
*λ*_2_	Center wavelength of the FBG2	1549.9954 nm
*λ*_3_	Center wavelength of the FBG3	1549.9698 nm
*P*_e_	Effective elastic optical coefficient of optical fiber	0.78
*E*	Elastic modulus of optical fiber	70 Gpa
*m*	Weight of mass ball	85 g
*d*	Diameter of fiber	125 μm
*g*	Gravitational acceleration	9.8 m/s^2^

**Table 2:** Partial comparison between the measurement results of the CFBG-FP sensor for heading angle detection vs. the reference angle.

	Reference Angle (Deg)	Roll Angle (Deg)	Pitch Angle (Deg)	Heading Angle (Deg)	Angle Deviation (Deg)
Northwest	45.0	4.0	0	46.2	1.2
	35.0	6.0	0	36.3	1.3
	25.0	0	0	24.9	0.1
	15.0	0	0	15.1	0.1
	5.0	0	0	5.4	0.4
Northeast	5.0	8.0	−1.0	7.3	2.3
	15.0	8.0	−2.0	17.0	2.0
	25.0	8.0	−2.0	26.1	1.1
	35.0	0	0	35.1	0.1
	45.0	0	0	44.5	0.5
	55.0	0	0	55.0	0
	65.0	5.0	5.0	64.2	0.8
	75.0	8.0	−2.0	76.3	1.3
	85.0	1.0	8.0	81.8	3.2
Southeast	85.0	5.0	5.0	84.1	0.9

## Data Availability

Data available upon request.
